# PHDMF: A Flexible and Scalable Personal Health Data Management Framework Based on Blockchain Technology

**DOI:** 10.3389/fgene.2022.877870

**Published:** 2022-04-13

**Authors:** Liangxiao Ma, Yongxiang Liao, Haiwei Fan, Xianfeng Zheng, Jintao Zhao, Ziyi Xiao, Guangyong Zheng, Yun Xiong

**Affiliations:** ^1^ Chinese Academy of Sciences Key Laboratory of Computational Biology, Bio-Med Big Data Center, Shanghai Institute of Nutrition and Health, University of Chinese Academy of Sciences, Chinese Academy of Sciences, Shanghai, China; ^2^ Shanghai Key Laboratory of Data Science, School of Computer Science, Fudan University, Shanghai, China; ^3^ Shanghai Clinical Research and Trial Center, Shanghai, China; ^4^ New York University Shanghai, Shanghai, China; ^5^ Peng Cheng Laboratory, Shenzhen, China

**Keywords:** personal health data, blockchain, smart contract, data provenance, data sharing

## Abstract

Currently, most of the personal health data (PHD) are managed and stored separately by individual medical institutions. When these data need to be shared, they must be transferred to a trusted management center and approved by data owners through the third-party endorsement technology. Therefore, it is difficult for personal health data to be shared and circulated over multiple medical institutions. On the other hand, the use of directly exchanging and sharing the original data has become inconsistent with the data rapid growth of medical institutions because of the need of massive data transferring across agencies. In order to secure sharing and managing the mass personal health data generated by various medical institutions, a federal personal health data management framework (PHDMF, https://hvic.biosino.org/PHDMF) has been developed, which had the following advantages: 1) the blockchain technology was used to establish a data consortium over multiple medical institutions, which could provide a flexible and scalable technical solution for member extension and solve the problem of third-party endorsement during data sharing; 2) using data distributed storage technology, personal health data could be majorly stored in their original medical institutions, and the massive data transferring process was of no further use, which could match up with the data rapid growth of these institutions; 3) the distributed ledger technology was utilized to record the hash value of data, given the anti-tampering feature of the technology, malicious modification of data could be identified by comparing the hash value; 4) the smart contract technology was introduced to manage users’ access and operation of data, which made the data transaction process traceable and solved the problem of data provenance; and 5) a trusted computing environment was provided for meta-analysis with statistic information instead of original data, the trusted computing environment could be further applied to more health data, such as genome sequencing data, protein expression data, and metabolic profile data through combining the federated learning and blockchain technology. In summary, the framework provides a convenient, secure, and trusted environment for health data supervision and circulation, which facilitate the consortium establish over medical institutions and help achieve the value of data sharing and mining.

## Introduction

With the development of information technology, personal health data (PHD) have started their transformation from a paper copy version to an electronic recording form. Currently, many personal health data are managed and transformed into electronic data in individual medical institutions, from where they must be transferred to a trusted central data management agency when need to be shared. Then, an authorization process based on third-party endorsement should be conducted before the original data being shared. Therefore, it is difficult to share personal health data among multiple medical institutions. In recent years, the rapid development of blockchain technology has provided us with a solution for personal health data storage and supervision without third-party endorsement.

Performing as an incorruptible and traceable distributed ledger, blockchain technology was first mentioned and practiced in Bitcoin (Nakamoto, 2009). Blocks are linked by hashing algorithms, so the original chain structure would get destroyed once any data in any block has been tampered with. In practice, the public blockchain and consortium blockchain are usually used for multi-party’s data supervision, while the former allows anyone to join the blockchain and the latter only permits authorized members to participate in the blockchain. For example, Bitcoin and Ethereum (Buterin, 2015) allow anyone or any organization to act as a blockchain node with reading and writing permission, while Hyperledger Fabric (Androulaki et al., 2018) allows only the recognized members to act as the blockchain nodes. The decentralization of public blockchain is achieved using the consensus algorithm of Byzantine fault tolerance (Lamport et al., 1982), which is applied in fields such as proof-of-work (PoW) (Dwork and Naor, 1993) and proof-of-stock (PoS) (King and Nadal, 2012); while for consortium blockchain, the Byzantine fault tolerance consensus algorithm is used together with the crash tolerance consensus algorithm such as raft (Ongaro and Ousterhout, 2014). Many public blockchains, known as blockchain 1.0, such as Bitcoin does not support smart contracts; instead, they are restricted in the “mining” of cryptocurrencies; therefore, coupled with the lack of regulation and the electricity resources wastes, governments from various countries have already shown their resistance to such blockchains. In addition to the “mining”, Ethereum and other public blockchains that support smart contracts, known as blockchain 2.0, are utilized in some decentralized financial applications (Hofman, 2017; Du et al., 2020). The blockchain 2.0 is limited to the financial field since its public nature worries many enterprises. The blockchain 3.0, supporting smart contract and federal organization, such as Hyperledger Fabric, has been widely used in the fields of finance, healthcare, judiciary, and logistic industries (Azaria et al., 2016; Ahmad et al., 2021; Li et al., 2021; Tao and Ling, 2021).

Due to the full disclosure nature of public blockchain, it is not suitable for supervision of personal health data; instead, an encryption algorithm is needed to guarantee data privacy and security. Meanwhile, the extremely low throughput of public blockchain also limits its application in health fields, for instance, the maximum throughput of Bitcoin is 7tps (Croman et al., 2016), and 15tps for Ethereum (Wang et al., 2019). Yue et al. (2016) have proposed the healthcare data gateway (HGD) that uses the consortium blockchain framework to store data; only specific personnel are granted access to the data, and patients would be able to manage their own personal health data as well. Griggs et al. (2018) fulfilled the real-time tracking and updating patients’ health data through applying the private blockchain framework coupled with remote medical sensor technology. Li et al. (2018) proposed a blockchain-based data preservation system (DPS) for medical data, which ensures the primitiveness and verifiability of stored data with the blockchain technology and secures data privacy with encryption algorithms. Ahram et al. (2017) constructed a protected health information system (PHI) called HealthChain, which realizes data scalable extension and privacy ensurance based on the Hyperledger Fabric permission network and smart contracts. Dagher et al. (2018) developed a PHI system called Ancile on the basis of Ethereum to achieve data access control and privacy security, with more attention attached to data sharing between owners and users. Ivan (2016) used public blockchain to store encrypted personal health data, in which data can be freely accessed and monitored by patients. Chen et al. (2018) combined blockchain with cloud services for managing and sharing personal health data. Wang et al. (2018) established a personal health data blockchain framework based on parallel execution to model and represent patients’ health and to analyze corresponding therapeutic regimens and clinical recommendations through computation. Azaria et al. (2016) proposed MedRec, a decentralized record management system to handle electronic medical records (EMRs), in which patients can access information from different medical institutions through its proof-of-work consensus algorithm. Jiang et al. (2018) offered a healthcare information exchange (HIE) platform called BlocHIE that uses two loosely coupled blockchains to handle electronic medical records and personal health data, with the combination of off-chain storage and on-chain verification to satisfy requirement of privacy and authorization. Zhang et al. (2018) built up an FHIRChain-based (Fast Healthcare Interoperability Resources) decentralized app, using digital health identities to authenticate participants. This app allows users to share specific and structured pieces of information rather than the entire document, so that the granularity level of shared data would decrease, and the readability of data and flexibility of sharing are improved. Xia et al. (2017) provided a blockchain-based system named MedShare, which solves the problem of health data sharing in the untrusted environment by employing smart contracts for data access control and provenance auditing.

The applications of blockchain technology mentioned above mostly focus on data privacy, security, and sharing. In these applications, the sharing processes are usually conducted through exchanging original health data, such as how Ancile sends health data to the users through HTTPS. Even though FHIRChain decreases the granularity level of data in which pieces of information could be sent partially and selectively, and the concern of data breaches still exists due to the inadequate supervision during the sharing process. Additionally, considering the rapid increase in the quantity of personal health data held by individual medical institutions, the mechanism of sharing original data has become unable to support the consortium system due to the ever-increasing amount of data exchanging across agencies. To overcome difficulties of health data supervision and circulation, we designed and developed a flexible and scalable personal health data management framework (PHDMF, https://hvic.biosino.org/PHDMF). The framework adopted consortium blockchain over multiple medical institutions, which offered the channel for more institutions to join the system in virtue of its nature of scalability. Additionally, the framework could guarantee personal health data security due to its exclusiveness to parties that were not involved in the blockchain, which solved the problem of data supervision in the circulation of the multi-party data-sharing process. Finally, a trusted computing environment was provided by the framework, in which data sharing with a meta-analysis could be performed by applying statistic information data instead of original data. The framework provides a convenient, secure, and trusted environment for health data exchange and circulation, which helps achieve the value of data sharing and mining.

## Materials and Methods

### Framework Design

**FIGURE 1:**
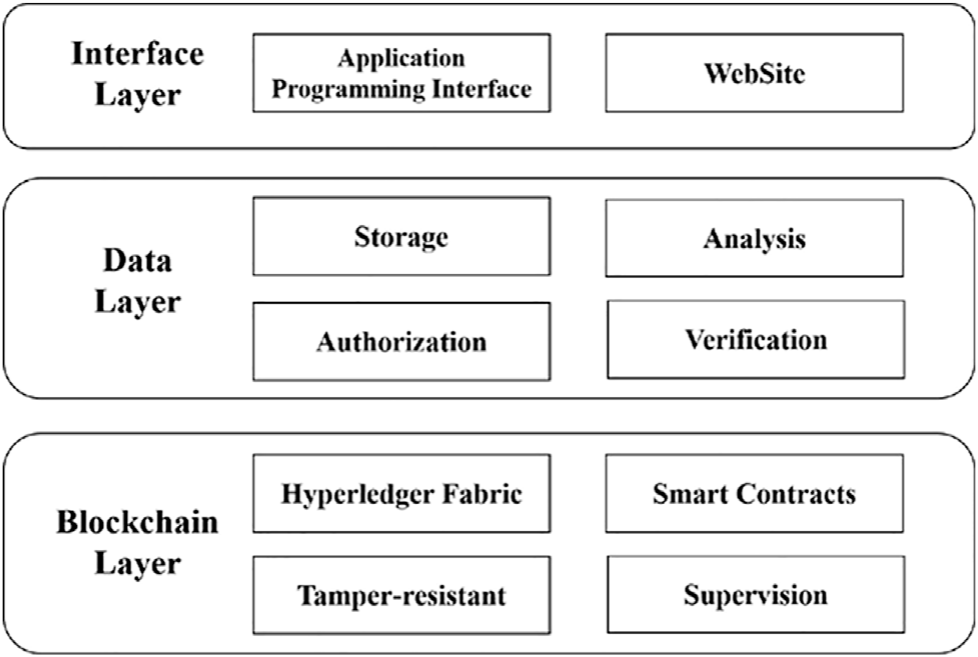
Structure of the personal health data management framework. The framework was composed of interface layer, data layer, and blockchain layer.

The personal health data management framework (PHDMF) was designed as a federal system based on consortium blockchain technology, which allowed the authorization, supervision, and modification of personal health data and provided a multi-party data sharing and mining solution as well (Figure 1). The interface layer provided the website and application programming interface (API) for users communicating with the system; the data layer consisted of local node servers and central servers, while the local node servers performing as the distributed storage scheme for personal health data of multi-party medical institutions, and the central servers offering data transaction management and statistic computation in a trusted environment; therefore, after the authorization of the data owner, statistic data from consortium participants could be collected for aggregate statistical analysis; the blockchain layer was designed as an infrastructure on the basis of the Hyperledger Fabric platform for recording the process of data authorization, operation, and modification.

**FIGURE 2:**
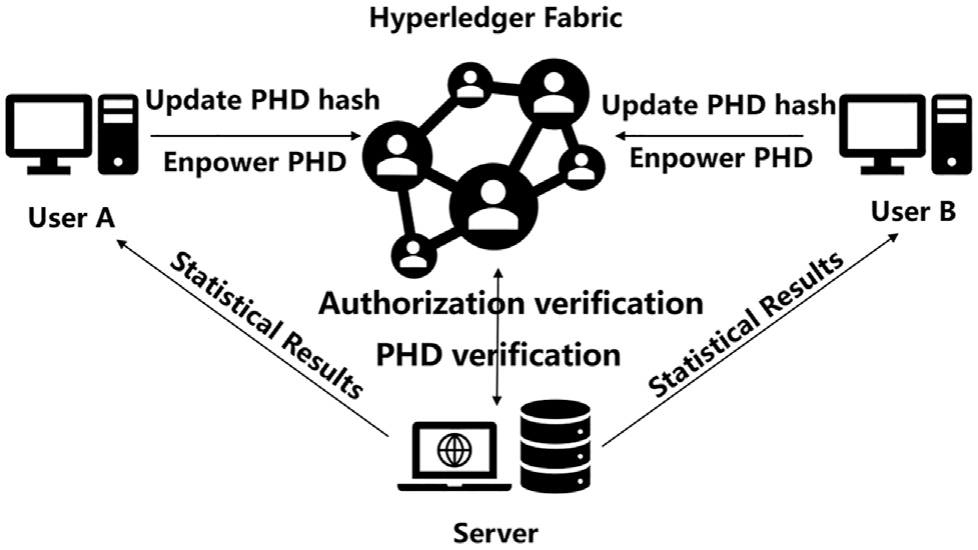
Physical implementation of the PHDMF. The framework consisted of the central servers, local node servers, and underlying blockchain.

In the system, an off-chain storage and on-chain verification combining strategy was adopted for personal health data storage and supervision (Figure 2). When data owners wanted to release their health data in the consortium, the hash value of health data would be calculated and recorded on the blockchain. Meanwhile, the original health data would be stored on local node servers. Then, data owners should verify the hash value of original data whether it was consistent with that on the blockchain in order to make tamper-resistant data. The adoption of an off-chain storage and on-chain verification combining strategy made the massive data transferring process being of no further use in data sharing. In practice, the local servers provided both data storage access and permission authorization interface. Data storage and access behaviors included data operation of upload, iteration, modification, download, and statistical analysis; permission authorization behaviors included the applying and processing of the permission request. The central servers provided three types of functions, namely, account management, authorization verification, and data verification and computation. Account management included account registration, log in, tracking, modification, and connection test; authorization verification offered a verifying mechanism for permission authorization of the whole system; data verification and computation implemented data hash value comparison and multi-party’s information data statistical calculation. The blockchain component offered a block generation mechanism of smart contract for data operation recording and a block information query and revise managements.

### System Implementation

The PHDMF adopted a front-end and back-end separation architecture. In detail, some webpage technologies such as HTML, CSS, and Vue were used for the front-end, while the Flask and Hyperledger Fabric platform were utilized for the back-end transaction handing and federal organization.

Vue has responsive programming and componentization features, and it possesses advantages including lightweight framework, simplicity, two-way data binding, componentization, separation of data and structure, virtual DOM, and fast running speed. Performing as a single-page application, Vue allows partial refresh of the page, so no request of all data and DOM are required for every redirection, access speed as well as user experience could be improved, and development time could be saved because of the third-party UI library.

Flask has the advantage of handiness, simplicity, and strong expansibility. Wide options for third-party libraries are also available, which together with the rich Python data analysis and machine learning libraries could provide the future development of the system with strong expansibility.

Hyperledger Fabric is the first open-source distributed ledger platform for enterprise application scenarios. Led by the Linux Foundation and founded by 30 initial business members including IBM, Hyperledger Fabric has a good open-source community. Fabric introduces permission management and supports dynamic node scaling and thus could serve as a technical solution for a flexible and scalable consortium blockchain.

## Results

### Applying for Becoming a Member of the Health Data Consortium

**FIGURE 3:**
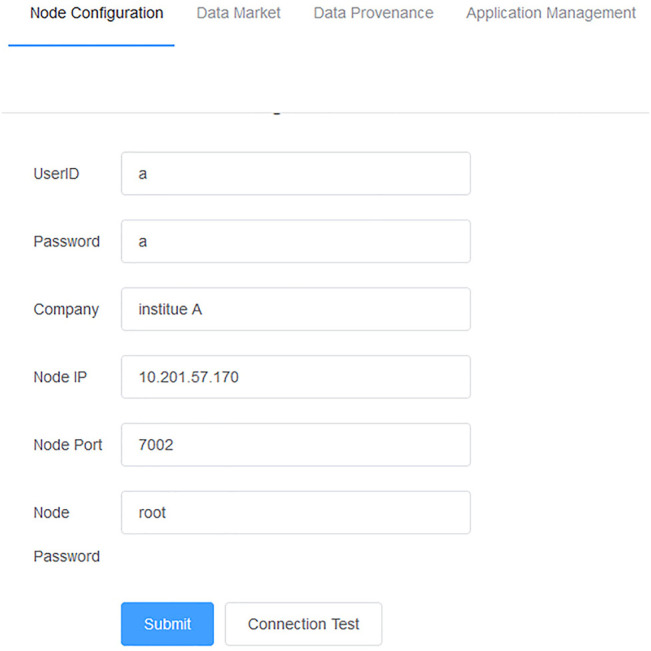
Node configuration of the PHDMF. Consortium members should configure the blockchain parameters within the framework.

The personal health data management framework (PHDMF) was designed to support a federal data consortium, which provided a flexible and scalable technical solution for member extension. In practice, when a user of medical institute wanted to become a new member of the data consortium, one should submit a participant application form to the management agency of the consortium first. After being approved by the consortium, one should download node client software of the framework. Then, one should install the software and configure blockchain parameters according to user guidance of the framework so that the new node could communicate with other nodes of the consortium correctly (Figure 3). Finally, one could store personal health data in local servers and release these data within the framework.

### Data Release and Storage for Consortium Members

**FIGURE 4:**
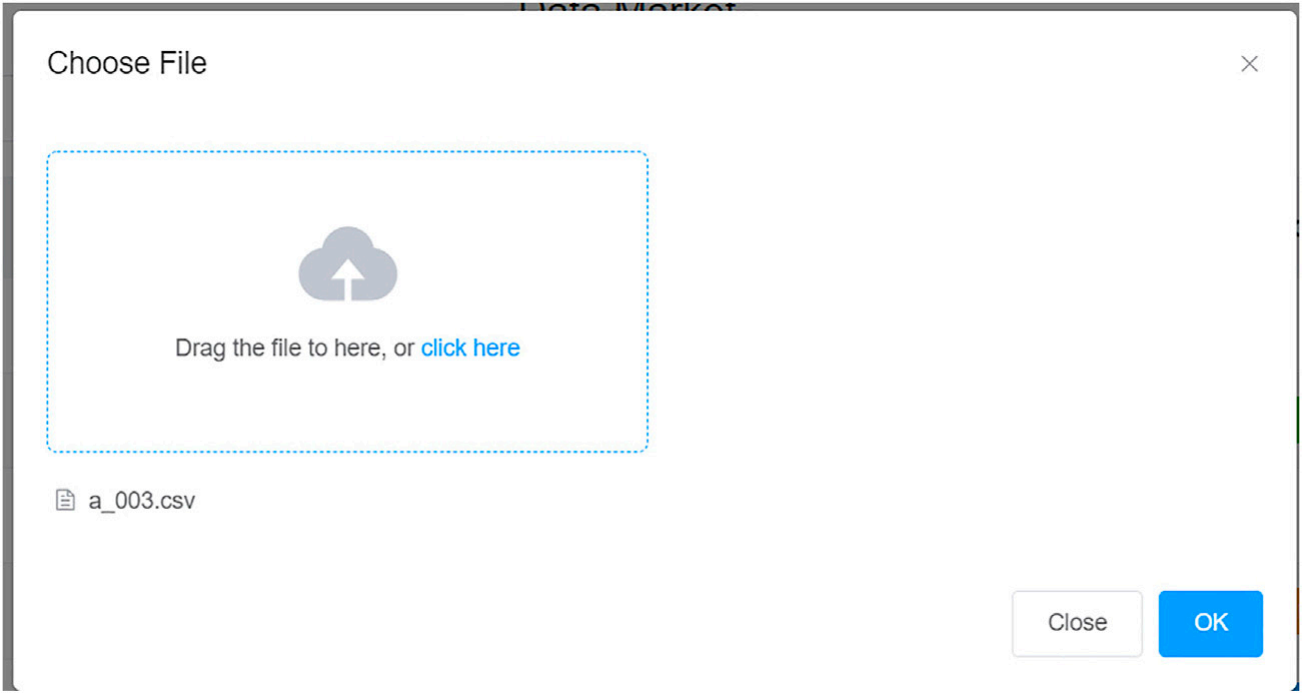
Data storage and release for consortium members. The framework adopted a distributed storage system, with massive personal health data mainly stored in the local server of consortium members.

In PHDMF, the strategy of data off-chain storage and on-chain verification reduced the storage space and waived the data key requirement for local servers, which was conducive to the expansion of the consortium. Data of consortium members could be released and protected securely by employing a distributed storage system, and the consistency of the hash value between stored data and blockchain records ensured the integrity and reliability of shared data. In practice, members of the consortium could upload their local personal health data using the graphic tool under the data mart of PHDMF. While the hash value of the uploaded health data would be recorded on the blockchain, the original health data would still be stored in the local storage space (Figure 4). After data being released in the PHDMF, data owners could configure access permission for these data within the consortium.

### Data Permission Configuration and Authorization in the Consortium

**FIGURE 5:**
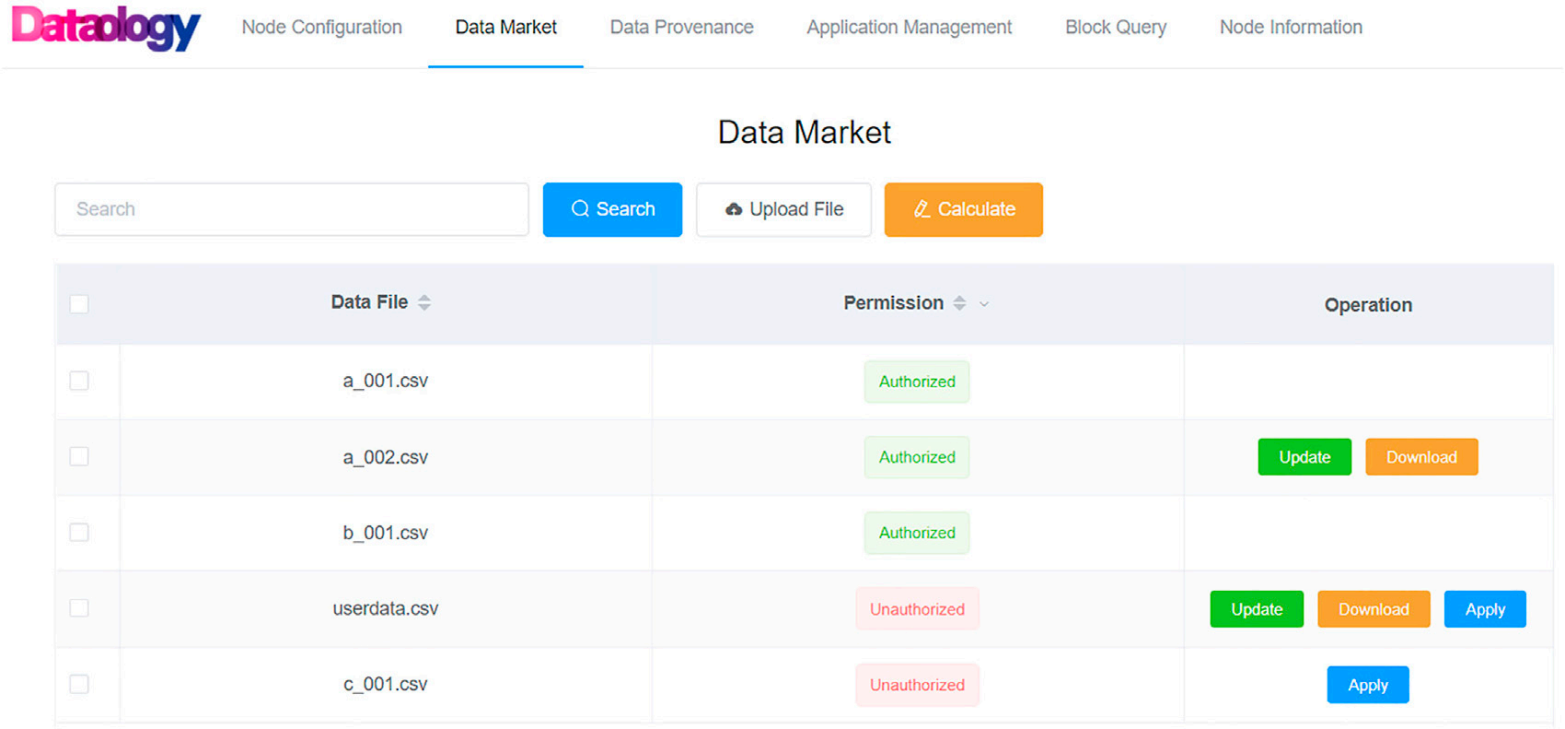
Personal health data permission configuration. Data owners could assign allowing or denying access permission to a specific dataset. Third-party users could apply for access permission to a released dataset.

**FIGURE 6:**
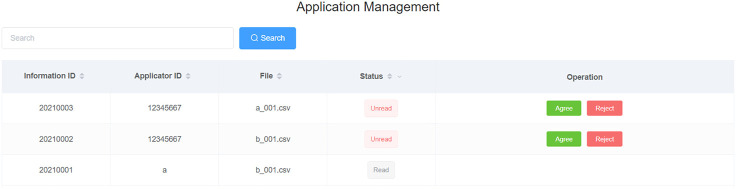
Personal health data permission authorization. Data owners could grant or deny access to third-party users through the smart contract.

The permission configuration allowed data owners to set permission (allow access or deny access) for their published data in the data mart of PHDMF. Third-party users could apply for access permission to public data released by the consortium members and are only allowed to use the data after being authorized by the data owners (Figure 5). Data owners could grant access to third-party users through the smart contract (Figure 6). In practice, third-party users could browse data released in the data mart of PHDMF; then, they needed to apply for access permission to interested data. After that, the data owners would receive the application and could either allow or deny access requests. The smart contract recorded processing of each application for permission and authorization, thus implementing data management and provenance.

### Data Provenance on the Consortium Blockchain

The blockchain recorded data operations such as upload, update, delete, authorization, and query in a distributed ledger manner. In detail, smart contracts were applied to transparently store and record data transactions and thus provided data provenance traceability for the consortium.

**FIGURE 7:**
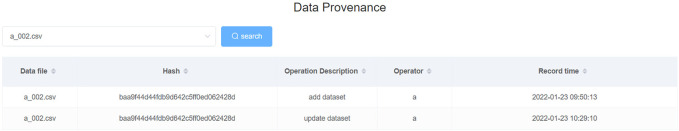
Data provenance on the consortium blockchain. Data provenance could be carried out through referring to dataset files, hash values, operation description, operators, time, and other information stored on the blockchain.

As shown in Figure 7, operations including data upload and update were involved in the process of owner-released health data on the PHDMF system. The blockchain recorded the dataset file, hash value, operation description, operator, time, and other information of the operation in an anti-tampered manner. Data owners and third-party users could query and browse the operation records through data provenance of the PHDMF system to ensure data security in the consortium. Moreover, users of the PHDMF system could browse consortium members’ information (node of distributed ledgers) through the node information menu, which described the detailed information of federal participants.

### Central Trusted Computing Environment and Data Statistical Analysis

**FIGURE 8:**
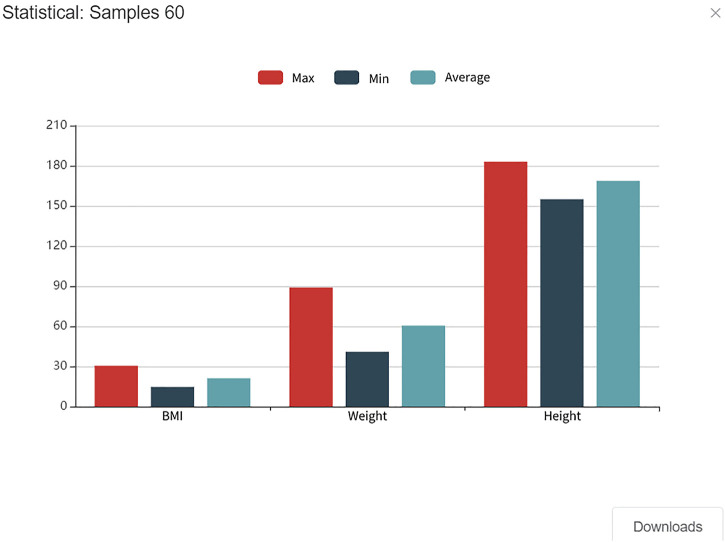
Aggregate statistical result for personal health data. Data from multiple parties would be delivered to the trusted computing environment on a central server in which aggregate statistical analysis would be performed.

For data sharing, the Ancile platform (Dagher et al., 2018) transmits complete user’s health data through HTTPS protocol, while FHIRChain (Zhang et al., 2018) shares data that are more fine-grained, and also the personal health data sharing on related medical blockchain is the whole original data. Nevertheless, such a sharing channel would require methods such as user agreements or electronic contracts to prevent data secondary sharing, which will be difficult to achieve. Even though it is possible to trace data records on the blockchain, it is hard to ensure the rights and interests of data owners. Here, we provided a new data-sharing technical solution in the PHDMF system, in which a central trusted computing environment for data exchange was offered. In practice, a central server was applied to build up a trusted environment for data collection and computation. First, third-party users would apply access permission to interested datasets released by the members of PHDMF. After authorization of dataset’s owners, statistic information data of these datasets instead of original health data were delivered to the trusted computing environment of the central server, in which an aggregate statistical analysis was performed. Finally, third-party users could obtain analytical results of multi-party datasets without granting the right to access original health data. Except for statistic methods, such a solution could be further applied in federated learning approaches. This data-sharing solution could greatly protect the rights and interests of dataset owners and provide third-party users with the expected outcome without compromising data security. As shown in Figure 8, third-party users could select multiple health datasets for aggregate analysis, and then statistical results of physiological indexes were presented in the form of bar charts, including sample number, sample maximum, sample minimum, and sample mean.

## Discussion

In this study, we built up a healthcare federal framework in the concern about data management and circulation based on the blockchain technology, which could ensure data security in the sharing process without the involvement of a third-party endorsement. In the blockchain layer of the framework, some mature cryptographic algorithms were adopted to make recorded data tamper resistant. Meanwhile, data provenance was guaranteed through recording every data operation and transaction by smart contracts. Additionally, an application of on-chain and off-chain combination architecture could effectively reduce the storage space required and waive the need of data keys, which benefited the scalability of the consortium. Finally, a data-sharing prototype was provided in the framework and that data sharing and aggregate statistical analysis could be performed without sharing the original data. During the analysis process, the third-party users could only read the statistical results but not download the original data; therefore, data from multiple parties can be shared for analysis purposes without having its original contents leaked. Such a data-sharing prototype could be further applied to more health data, such as genome sequencing data, protein expression data, metabolic profile data with the federated learning and the blockchain technology.

There are some drawbacks of the framework which should be optimized in future. First, the single-customer transaction throughput of the framework (based on the Hyperledger Fabric platform) reaches hundreds of times per second currently; however, such processing speed is not compatible with the future data expansion. Therefore, better strategies and algorithms should be designed to improve the transaction throughput of the framework. Second, security of the framework needs more improvements because the current encryption algorithm of the blockchain such as RSA may not be able to provide sufficient security faced with the quantum computing technology. Last, more comprehensive management strategies are needed to prevent smart contracts from developing vulnerability. Smart contracts of the framework are applied in a transparent and explicit manner, which is easy to be attacked by a computer virus. Therefore, a more secure strategy for smart contracts should be developed in future for the framework.

## Data Availability

Publicly available datasets were analyzed in this study. These data can be found at: https://hvic.biosino.org/PHDMF.
